# Tau-Mediated Nuclear Depletion and Cytoplasmic Accumulation of SFPQ in Alzheimer's and Pick's Disease

**DOI:** 10.1371/journal.pone.0035678

**Published:** 2012-04-25

**Authors:** Yazi Ke, Joe Dramiga, Ulrich Schütz, Jillian J. Kril, Lars M. Ittner, Hannsjörg Schröder, Jürgen Götz

**Affiliations:** 1 Alzheimer's and Parkinson's Disease Laboratory, Brain & Mind Research Institute, University of Sydney, Camperdown, New South Wales, Australia; 2 Department II of Anatomy and Neuroanatomy, University of Cologne, Cologne, Germany; 3 Center for Molecular Medicine Cologne, CMMC, University of Cologne, Cologne, Germany; 4 Disciplines of Medicine and Pathology, University of Sydney, Sydney, New South Wales, Australia; Thomas Jefferson University, United States of America

## Abstract

Tau dysfunction characterizes neurodegenerative diseases such as Alzheimer's disease (AD) and frontotemporal lobar degeneration (FTLD). Here, we performed an unbiased SAGE (serial analysis of gene expression) of differentially expressed mRNAs in the amygdala of transgenic pR5 mice that express human tau carrying the P301L mutation previously identified in familial cases of FTLD. SAGE identified 29 deregulated transcripts including *Sfpq* that encodes a nuclear factor implicated in the splicing and regulation of gene expression. To assess the relevance for human disease we analyzed brains from AD, Pick's disease (PiD, a form of FTLD), and control cases. Strikingly, in AD and PiD, both dementias with a tau pathology, affected brain areas showed a virtually complete nuclear depletion of SFPQ in both neurons and astrocytes, along with cytoplasmic accumulation. Accordingly, neurons harboring either AD tangles or Pick bodies were also depleted of SFPQ. Immunoblot analysis of human entorhinal cortex samples revealed reduced SFPQ levels with advanced Braak stages suggesting that the SFPQ pathology may progress together with the tau pathology in AD. To determine a causal role for tau, we stably expressed both wild-type and P301L human tau in human SH-SY5Y neuroblastoma cells, an established cell culture model of tau pathology. The cells were differentiated by two independent methods, mitomycin C-mediated cell cycle arrest or neuronal differentiation with retinoic acid. Confocal microscopy revealed that SFPQ was confined to nuclei in non-transfected wild-type cells, whereas in wild-type and P301L tau over-expressing cells, irrespective of the differentiation method, it formed aggregates in the cytoplasm, suggesting that pathogenic tau drives SFPQ pathology in post-mitotic cells. Our findings add SFPQ to a growing list of transcription factors with an altered nucleo-cytoplasmic distribution under neurodegenerative conditions.

## Introduction

Alzheimer's disease (AD) is characterized by both amyloid-β (Aβ) plaques and tau tangles in the brain while tau pathology in the absence of plaques occurs in a subset of frontotemporal lobar degeneration (FTLD-Tau) that includes FTDP-17 and Pick's disease (PiD) [1]. Features of FTLD-Tau have been reproduced in transgenic mice expressing FTDP-17 mutant tau [2]: P301L tau transgenic pR5 mice are characterized by tau hyperphosphorylation, tangle formation in the amygdala and hippocampus, and memory impairment [3].

To determine the consequences of tau pathology, both in animal models and human disease, we and others have applied the tools of functional genomics [4]. Proteomic analysis, e.g., revealed separate and synergistic modes of Aβ and tau on mitochondrial functions [5], [6] while in a transcriptomic study, we identified the detoxifying enzyme glyoxalase I as a target of tau toxicity [7]. These studies were all done with total brain, while here we focused on the amygdala, a brain area with prominent tau pathology and affected early on in AD pathogenesis. Furthermore, instead of using gene arrays to identify differential gene expression, we used the unbiased, though less frequently applied SAGE (Serial Analysis of Gene Expression) method [8].

We identified 29 differentially expressed genes in pR5 transgenic amygdala, of which 11 were up- and 18 down-regulated compared to non-transgenic controls. Among these was *Sfpq* that encodes a nuclear splicing factor and transcriptional regulator. Our subsequent analysis for the first time revealed a nucleo-cytoplasmic redistribution of SFPQ under pathological conditions, similar to what has been reported for TDP-43 that forms cytoplasmic aggregates in amytrophic lateral sclerosis (ALS) and FTLD-TDP [9], and FUS in ALS [10], [11] and FTLD-FUS [12]. This highlights the nucleo-cytoplasmic redistribution of transcription factors as a prominent pathomechanism in neurodegeneration. Our data suggest that pathological tau may cause neuronal dysfunction, at least in part, by mislocalizing proteins such as those implicated in mRNA processing and/or splicing.

## Results

### SAGE analysis of P301L tau-expressing mice

**Figure 1:**
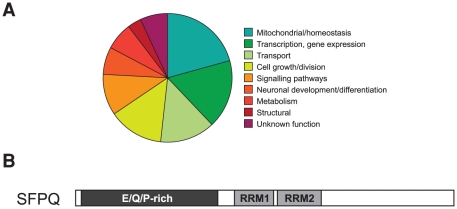
Deregulation of the nuclear factor *Sfpq* in tau transgenic mice. (A) Transcriptomic SAGE analysis of P301L tau mutant pR5 (TG) compared to wild-type (WT) amygdala identified differentially expressed genes within several functional categories (pie chart). The strongly deregulated ‘transcription’ genes (green) included *Sfpq*. (B) Scheme of the domain structure of the 707 amino acid-long nuclear protein SFPQ. The amino-terminal glutamic acid (E)/glutamine(Q)/proline(P)-rich domain is followed by two RNA/DNA-binding domains (RRMs).

In P301L mutant tau transgenic pR5 mice, NFT formation is initiated in the amygdala [13], [14]. To determine differentially regulated genes in pR5 mice compared to non-transgenic control littermates, we isolated mRNAs from dissected amygdalae and performed an unbiased SAGE analysis. We obtained 92,000 sequenced tags (46,586/wild-type and 46,905/pR5), which allowed us to identify differentially expressed genes (**Table 1**). By disregarding repetitive elements and SAGE linkers, most of the mitochondrial genes in the library showed a reduced expression in the transgenic sample. This included both mitochondrial and nuclear encoded mitochondrial genes. Specifically, subunits 6 and 8 of ATP synthase F0 (*mt-Atp6* and mt-*Atp8*) as well as subunit 3 of Cytochrome c oxidase (*mt-Co3*) were down-regulated, while subunit 1 of Cytochrome c oxidase (*mt-Co1*) was up-regulated. These genes are encoded by the mitochondrial genome. The nuclear encoded mitochondrial genes included isoforms 1 and 3 of the ATP synthase subunit c (*Atp5g1* and A*tp5g3*, respectively). While in the wild-type sample, isoform 1 yielded 7 tags, in pR5 it yielded none; isoform 1 yielded 42 tags in wild-type and only 17 in pR5. This deregulation may contribute to the reduced mitochondrial activity that characterizes pR5 mice as shown by us previously, both at the level of the proteome and functionally [5], [15]. In addition to mitochondrial genes, 24 genes were identified that reached a significance of p<0.01 of differential regulation (**Table 1**). Of these, 9 were up- and 14 down-regulated. They included genes with functions in mitochondrial/homeostasis, transcription/gene expression, transport, cell growth/division, signaling pathways, and others (**Fig. 1A**).

**Table 1:** SAGE (Serial Analysis of Gene Expression) analysis in the amygdala of P301L tau mutant pR5 mice identifies 29 deregulated genes that includes *Sfpq*.

#	Gene name	Tag	WT	P301L	P value	Unigene
1	Sfpq, Splicing factor proline/glutamine rich	combined	0.0	8.0	0.0041	Mm.277094
2	Zranb1, Zinc finger, RAN-binding domain 1	AGGAGATGGAG	0.0	7.0	0.0079	Mm.389984
3	XAP5 protein	TTGGAGCTGGA	0.0	7.0	0.0079	Mm.4370
4	Enpp2, ectonucleotide pyrophosphodiesterase 2	combined	4.0	16.0	0.0076	Mm.250256
5	Sh3glb2, SH3-domain GRB2-like endophilin B2	combined	6.0	22.9	0.0015	Mm.33343
6	Gfra4, Glial cell line derived neurotrophic factor family receptor alpha 4	TGCACTGAGAA	5.0	17.9	0.0069	Mm.198399
7	Ttr, transthyretin	combined	20.1	49.8	0.0003	Mm.2108
8	Kcnk1, Potassium channel, subfamily K, member 1	GCAGATGGCAA	18.1	37.9	0.0081	Mm.10800
9	Ppp3cb, Protein phosphatase 3, catalytic subunit, beta isoform	combined	66.2	110.6	0.0008	Mm.274432
10	Itm2c, integral membrane protein 2C	GTAGTGGAGCC	75.3	119.6	0.0015	Mm.29870
**11**	**mt-Co1, Cytochrome c oxidase subunit I**	**GCTGCCCTCCC**	**1.0**	**12.0**	**0.0019**	**NA**
**12**	**mt-Co3, Cytochrome c oxidase, subunit III**	**ATACTGACATT**	**442.5**	**332.9**	**0.0001**	**NA**
**13**	**mt-Atp8, ATP synthase, F0 subunit 8**	**ATAATACATAA**	**868.0**	**652.8**	**3E-08**	**NA**
**14**	**Atp5g1, ATP synthase, F0 complex, isoform 1**	**CCAGTCCTGGT**	**42.1**	**16.9**	**0.0010**	**Mm.371547**
**15**	**Atp5g3, ATP synthase, F0 complex, isoform 3**	**GCAAACAAGAT**	**7.0**	**0.0**	**0.0076**	**Mm.2966**
16	Sepw1, selenoprotein W, muscle 1	TTTCCAGGTGT	99.3	62.8	0.0041	Mm.42829
17	Cplx2, Complexin-2	ATGACAAAGAA	59.2	31.9	0.0042	Mm.268902
18	Gtf2ird1, General transcription factor II repeat domain-containing 1	TAAGTGGAATA	26.1	10.0	0.0072	Mm.332735
19	Sept3, Septin 3	combined	41.1	19.9	0.0066	Mm.309707
20	Sept5, Septin 5	CTCCGTTTTGT	47.2	16.9	0.0001	Mm.20365
21	Mtpn, Myotrophin	TACATCCGAAT	18.1	5.0	0.0063	Mm.182746
22	Dbn1, Drebrin 1	GCAATAAATGG	29.1	8.0	0.0004	Mm.19016
23	Papola, poly (A) polymerase alpha	ACTGGAGTTTG	10.0	1.0	0.0062	Mm.255877
24	Pja1, praja1, RING-H2 motif containing	combined	10.0	1.0	0.0062	Mm.8211
25	Rpo1-4, RNA polymerase 1–4	GCTGGAACTGG	7.0	0.0	0.0076	Mm.135581
26	1700021K19Rik, RIKEN cDNA 1700021K19 gene	GTCATCTTTAA	7.0	0.0	0.0076	Mm.327319
27	H2afv, H2A histone family, member V	TGTTGATTGGC	7.0	0.0	0.0076	Mm.27624
28	Tmed8, Transmembrane emp24 domain containing 8	combined	7.0	0.0	0.0076	Mm.374912
29	Clk1, CDC-like kinase 1	GCCAAACCAAA	13.87	3.03	0.0085	Mm.1761

SAGE was used to obtain a total of 92,000 sequence tags from pooled amygdalae dissected from ten 10 month-old male pR5 mice (P301L) and ten wild-type (WT) littermate controls. Numbers of counted tags are listed for the two genotypes; ‘combined’ indicates that more than one tag was obtained per deregulated gene (for these, the individual tags are listed in Table 2). 29 genes presented a significant (p<0.01) regulation, including nuclear and mitochondrial encoded mitochondrial genes that are shown in light grey (Gene ID for mt-Co1: 17708; mt-Co3: 17710; mt-Atp8: 17706). Of these, 11 were up- (in white) and 14 down-regulated (in grey), with mitochondrial genes indicated (in **bold**).

### Deregulated genes

We identified a total of 29 genes that were significantly deregulated in pR5 amygdala (**Table 1**). Importantly, several of these have been implicated in neurodegenerative diseases other than AD or FTD, in particular those with functions in mitochondria. For example, among the up-regulated genes in pR5 amygdala is *Itm2c* that encodes an integral membrane protein. Genetic variants of ITMC2 have been associated with hemorrhagic stroke in humans [16]. *Ttr* encoding transthyretin is also up-regulated; gene mutations have been implicated in multiple forms of amyloid polyneuropathy, a disease characterized by systemic deposition of TTR amyloid [17], [18]. *Enpp2* is up-regulated; it encodes a phosphodiesterase also known by the name of autotaxin, that has a possible role in metastasis [19].

*Cplx2* is among the down-regulated genes in pR5 amygdala. It encodes complexin 2, an essential protein with a role in synaptic vesicle fusion [20]. Abnormal levels of CPLX2 have been implicated in Huntington's disease [21], while its levels are reduced in AD [22]. *Septin 3* and *5* are both down-regulated in pR5, with Septin 5 accumulating in Parkinson's disease (PD) brain [23]. Septins play important roles in many cellular processes by providing rigidity to the cell membrane, serving as scaffolds to recruit proteins to specific subcellular loci, and creating membrane diffusion barriers to establish discrete cellular domains [24]. Another down-regulated gene is *Pja1* that encodes the E3 ubiquitin ligase Praja that is expressed abundantly in brain and that has been proposed as a candidate gene in X-linked mental retardation (MRX) [25]. *Pja1* is up-regulated in the basolateral amygdala during the formation of fear condition memory [26], and interestingly, it is in the basolateral amygdala where pR5 mice specifically accumulate tau [27]. Finally, *Dbn1* is down-regulated in the pR5 amygdala. This gene encodes the actin-binding postsynaptic protein drebrin1 that regulates synaptic plasticity. Its levels are reduced in hippocampal synapses in AD [28], and its mRNA levels were found to negatively correlate with PHF-tau, i.e. abnormally phosphorylated tau [29].

### Deregulation of the gene encoding SFPQ

**Figure 2:**
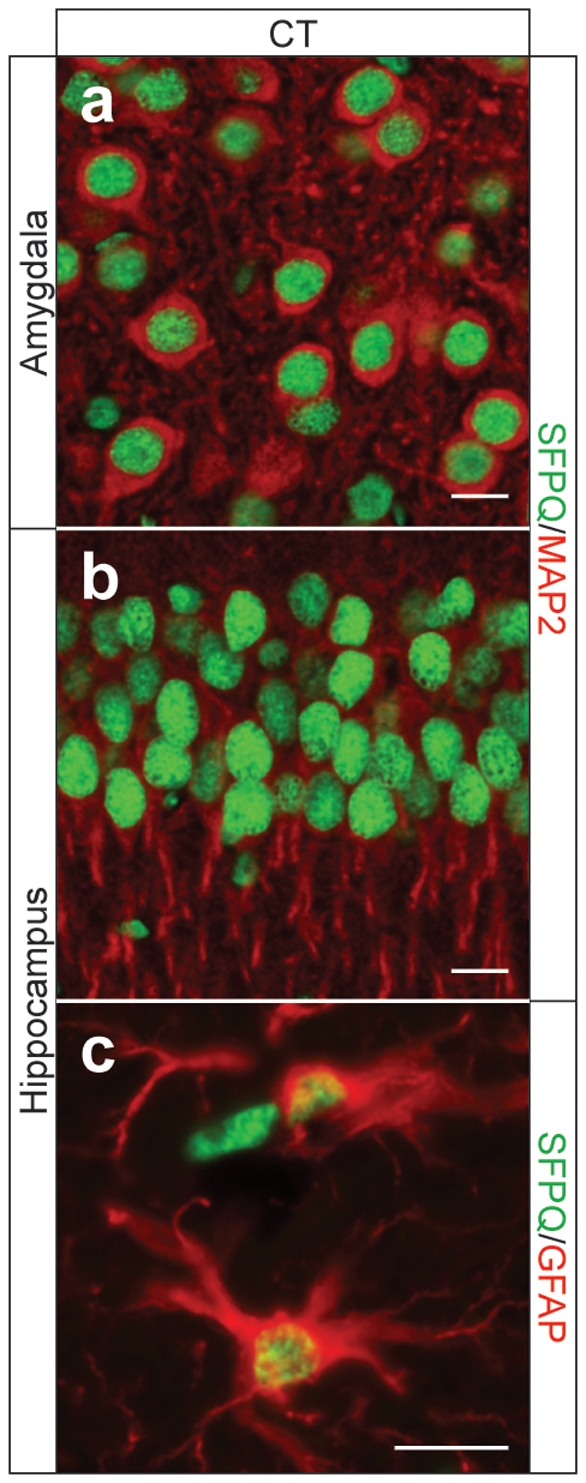
Neuronal and glial expression of SFPQ revealed in non-transgenic wild-type (CT) control mouse brain shown for the amygdala (A) and the hippocampus (B,C). Double immunofluorescence for SFPQ (green)/MAP2 (red) (A,B) and SFPQ (green)/GFAP (red) (C) reveals an exclusively nuclear localization in both neurons and astrocytes of WT mice.

While several genes were identified as being deregulated based on just one tag, for others, up to four tags were represented in the SAGE library, increasing the confidence in the finding of deregulation (**Table 2**). The latter group included *Sfpq* (splicing factor proline/glutamine rich) that encodes a large nuclear protein, SFPQ, implicated in cellular processes such as transcriptional regulation and RNA splicing. The protein is also known as PSF (Polypyrimidine tract-binding protein-associated Splicing Factor) [30]–[32]. The 707 amino acid-long nuclear protein SFPQ is highly conserved and contains an amino-terminal glutamic acid(E)/glutamine(Q)/proline(P)-rich domain followed by two RNA/DNA-binding domains (RRMs) (**Fig. 1B**). SFPQ is expressed by neurons and glia, and in both it is confined to nuclei (**Fig. 2**).

**Table 2:** List of SAGE tags of deregulated genes for which multiple tags were identified.

Gene name	Tag	WT	P301L	Ratio	P value	UniGene
Sfpq	combined	0	8,0	15,9	0,0041	Mm.277094
	TTGTGTGCTGT	0	3,0	6,0	0,1259	
	GGTCAGCTAAA	0	2,0	4,0	0,2512	
	CGTACTGAGCG	0	2,0	4,0	0,2512	
	ATCCACAGTCC	0	1,0	2,0	0,5012	
Enpp2	combined	4,0	16,0	4,0	0,0076	Mm.250256
	GTGCTGCCAGT	3,0	12,0	4,0	0,0219	
	AAGATGCACAC	1,0	4,0	4,0	0,2239	
Sh3glb2	combined	6,0	22,9	3,8	0,0015	Mm.295493
	GCTCTGGCTGG	6,0	20,9	3,5	0,0039	
	GATCCCGACTG	0	2,0	4,0	0,2512	
Ttr	combined	20,1	49,8	2,5	0,0003	Mm.2108
	TTCAAAAGCCC	2,0	2,0	−1,0	1	
	GAACGGGGAAA	1,0	10,0	9,9	0,0066	
	AATTCGCGGAT	17,1	37,9	2,2	0,0049	
Ppp3cb	combined	66,2	110,6	1,7	0,0008	Mm.24381
	TGCAAAGCTCC	1,0	1,0	−1,0	1	
	GGCCGCTGCTC	62,2	105,6	1,7	0,0008	
	GGCCGCTGCAA	3,0	4,0	1,3	0,7413	
Sept3	combined	41,1	19,9	−2,1	0,0066	Mm.309707
	TAGATGTTGCT	36,1	17,9	−2,0	0,0135	
	TACATTTTGCT	1,0	1,0	−1,0	1	
	GTGTACATACA	4,0	1,0	−4,0	0,2138	
Pja1	combined	10,0	1,0	−10,1	0,0062	Mm.8211
	TTCCCTCCCCC	1,0	0	−2,0	0,5012	
	GGTTAATGTTC	9,0	1,0	−9,1	0,0115	
Tmed8	combined	7,0	0	−14,1	0,0076	Mm.374912
	GGCTAAAATAA	6,0	0	−12,1	0,0151	
	ACCAGCTCTCA	1,0	0	−2,0	0,5012	

Significance was reached by combining the counts of multiple tags for the same gene.

### Nuclear depletion of SFPQ in AD and PiD brain

**Figure 3:**
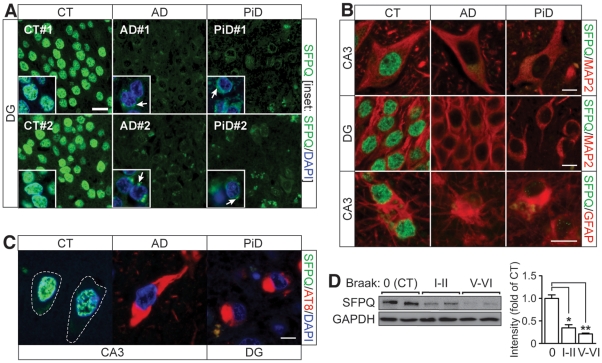
Nucleo-cytoplasmic redistribution of SFPQ in human disease. (A) Compared to controls (CT), SFPQ (green) is massively depleted from hippocampal nuclei in AD and PiD cases. It accumulates in patches (arrows) in the cytoplasm as shown by co-staining with nuclear DAPI (blue) at higher magnification (insets). (B) IF for SFPQ (green) and GFAP or MAP2 (red) reveals that in both AD and PiD brain, SFPQ is almost completely depleted from neuronal and astrocytic nuclei. (C) Neurons harboring AD tangles and PiD Pick bodies are similarly depleted of SFPQ (green) as shown by co-staining for phospho-tau (AT8, red) and DAPI (blue). (D) Immunoblot analysis of Braak stage 0 (CT), entorhinal stage I–II of AD pathology, and neocortical stage V–VI (terminal pathology) reveals massively reduced SFPQ levels as disease progresses (n = 6–8; *,p<0.01; **,p<0.001). Scale bars, 10 µm.

We next analyzed SFPQ in human diseases with tau pathology, by staining paraffin-embedded sections from the hippocampal formation of six sporadic AD cases, six sporadic PiD cases, and eight non-demented CT controls. SFPQ was completely depleted from most nuclei in AD and PiD hippocampi, and accumulated in focal patches in the cytoplasm, resembling aggregates (**Fig. 3A**). Interestingly, double immunofluorescence (IF) for SFPQ/GFAP or SFPQ/MAP2 revealed that in both AD and PiD brain, SFPQ was depleted from nuclei in both neurons and astrocytes (**Fig. 3B**). Accordingly, neurons harboring either AD tangles or PiD Pick bodies were also depleted of SFPQ as shown by co-staining for phospho-tau (AT8), SFPQ and DAPI (**Fig. 3C**). Immunoblot analysis of human entorhinal cortex samples with Braak stage 0 (CT), entorhinal stages I–II (minimal AD pathology) and neocortical stages V–VI (terminal pathology) revealed 65.3±6.7% reduced SFPQ levels for stages I–II, and a 78.7±1.9% reduction for V–VI compared with CT (**Fig. 3D**). This suggests that the SFPQ pathology may progress together with the tau pathology in AD.

### Tau induces cytoplasmic sequestration of SFPQ

**Figure 4:**
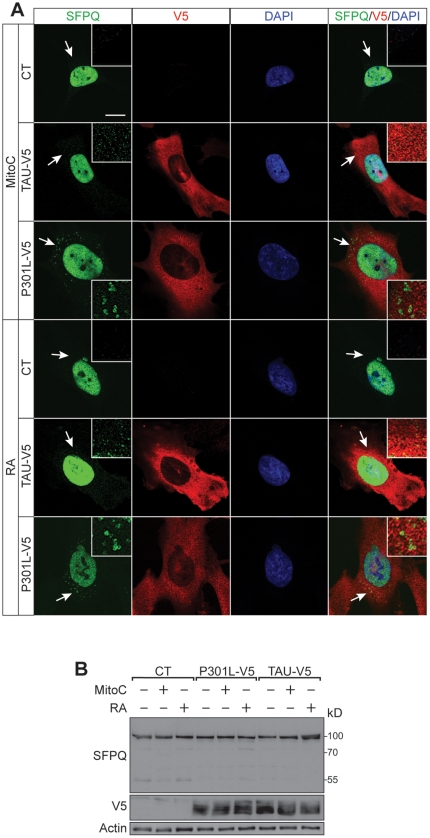
Tau transfection causes SFPQ aggregation in postmitotic cells. (A) Mitomycin C (Mito C)-mediated cell cycle arrest or neuronal differentiation with retinoic acid (RA) of V5-tagged wild-type or P301L tau-expressing SH-SY5Y compared to untransfected (CT) SH-SY5Y neuroblastoma cells reveals SFPQ aggregates (arrows) in the cytoplasm that are not seen in CT. Insets: detailed view of vesicular SFPQ in the cytoplasm. Nuclear staining: DAPI (blue). (B) Western blotting reveals that total levels of SFPQ are not altered under any of these conditions. Actin has been used for normalisation.

To determine the effects of pathological tau on SFPQ directly, we stably expressed both wild-type and P301L human tau in human SH-SY5Y neuroblastoma cells, a cell culture model of tau pathology [33], followed by either mitomycin C (Mito C)-mediated cell cycle arrest or neuronal differentiation with retinoic acid (RA). Confocal microscopy revealed that SFPQ was confined to nuclei in non-transfected wild-type cells, whereas in both wild-type and P301L tau-expressing cells, irrespective of the differentiation method, it formed vesicular aggregates in the cytoplasm (**Fig. 4A**). These were more pronounced in the mutant, and were remarkably similar to those identified in P301L tau-expressing cells. Within the sensitivity of the Western blot assay, total levels of SFPQ were not altered under any of these conditions (**Fig. 4B**). Together this suggests that pathogenic tau drives SFPQ pathology in post-mitotic cells. How tau causes the altered nucleo-cytoplasmic distribution of SFPQ remains to be determined.

## Discussion

By using SAGE as an unbiased transcriptomic approach we identified *Sfpq* as a downstream target of tau. We further found a nucleo-cytoplasmic redistribution of the nuclear protein SFPQ in AD and PiD pointing at a putative role for deregulated transcription factors in neurodegeneration. For both TDP-43 and FUS, a similar redistribution has been reported in ALS, and FTLD-TDP or FTD-FUS, respectively [9], [12]. SFPQ promotes neuronal survival in development [34], and increased SFPQ levels sensitize neurons to excitotoxic damage *in vitro* [35]. Hence, although tau itself may have nuclear functions [36], the nuclear depletion of SFPQ identified here suggests that pathological tau may cause neuronal dysfunction, at least in part, by mislocalizing proteins with nuclear functions. This extends the molecular targets of pathological tau beyond axons and dendritic processes [37], [38].

SFPQ is a transcriptional repressor of several genes, by inhibiting transcriptional co-activators or by binding to a consensus palindromic sequence (CTGAGTC) in the promoter region [39], [40]. Two of these sequences are found in the putative *Sfpq* promoter suggesting that SFPQ may repress its own transcription. SFPQ not only functions as a repressor of gene transcription, but it can also up-regulate gene expression by increasing mRNA stability as shown for the inducible *Cyclooxygenase-2* (*Cox-2*) gene [41].

Arresting P301L tau-expressing SH-SY5Y cells with either mitomycin C (mito C) or retinoic acid (RA), which causes a cytoplasmic accumulation of SFPQ in P301L tau-expressing cells and not, untransfected controls, rules out a direct role for mitosis in the altered localization of SFPQ. In addition, neurons are post-mitotic so the cytoplasmic localization found by us for SFPQ in AD and PiD brains cannot be due to mitosis. SFPQ redistribution could eventually induce cell death by contributing to apoptosis or mitosis, since a nuclear redistribution of SFPQ into aggregates has been observed in apoptotic cells, and a cytoplasmic redistribution in mitotic cells [42], [43]. It is therefore possible that stressed neurons try to cope with elevated tau levels by cytoplasmic sequestration of SFPQ.

Loss of nuclear SFPQ and/or its cytoplasmic accumulation may contribute to cellular degeneration also directly: The *Sfpq* homologue *whitesnake* is required for cell survival and normal brain development in zebrafish, where it is mostly detected in regions enriched with neuronal precursors: this suggests that an impairment in SFPQ function could contribute to neuronal cell death [34]. *Sfpq* overexpression *per se* may contribute to neuronal death since SFPQ sensitizes neurons to neurotransmitter-induced death *in vitro* [35]. Furthermore, studies in a cancer cell line identified SFPQ as a tumor suppressor suggesting that an altered distribution and depletion from nuclei could affect transcription of target genes [44].

Together, our finding of an aberrant cytoplasmic localisation of SFPQ in both AD and PiD brains and P301L tau-expressing SH-SY5Y neuroblastoma cells suggests a role for SFPQ in neurodegeneration with a tau pathology. Our study further links FTLD-Tau with the cytoplasmic aggregation of the nuclear protein SFPQ to FTLD-TDP with the cytoplasmic aggregation of the nuclear protein TDP-43 [45], [46], highlighting RNA mismanagement as a general pathomechanism [47].

## Materials and Methods

### Ethics statement

Human brain tissue was obtained from the Australian Brain Bank Network's Sydney Brain Bank, with approval from the Human Ethics Review Committee of the University of Sydney. Written informed consent was obtained from donors or donors next of kin for brain donation. The animal experiments were approved by the Animal Ethics Committee (AEC) of the University of Sydney and the Animal Care Committee of the University of Cologne (approval number K00/1-2009/3/4914).

### Transgenic mice

The pR5 mouse strain expresses the longest human tau isoform together with the FTLD-Tau (FTDP-17) pathogenic mutation P301L in neurons, with NFT formation occurring first in the amygdala and then in the hippocampus [13], [27], [48].

### RNA isolation

For the SAGE analysis, amygdalae were dissected from ten 10 month-old male pR5 mice and ten wild-type male littermate controls. At 5–6 months of age, NFT formation is initiated in the amygdala, with numbers increasing as the mice age. Phenotypically the mice show no obvious impairment, neither at 5 nor at 10 months of age [13], [14]. Brains were homogenized in Trizol (Invitrogen, Carlsbad, CA) to isolate high quality RNA, following the manufacturer's instructions. To remove contaminating DNA, the RNA was incubated with RNase-free DNase I (Promega, Wisconsin, MD) and quantified by UV spectroscopy at 260 nm. Between 10 and 30 µg of RNA was obtained from each amygdala, and RNA integrity was confirmed by gel electrophoresis using Bioanalyser chromatography (Agilent, Santa Clara, CA).

### Construction of the SAGE library

SAGE libraries were established by Memorec Stoffel GmbH (Cologne, Germany) as described [49], using 20 µg of total RNA pooled from the two genotypes. Enrichment of mRNA was achieved by affinity chromatography, using oligo-dT-coupled Dynabeads (Dynal, Invitrogen, Carlsbad, CA). In brief, mRNA was converted to cDNA using oligo-d(T)_25_ primers (Invitrogen) and Superscript II reverse transcriptase (Gibco BRL, Carlsbad, CA). The single-stranded cDNA was converted to double-stranded cDNA and digested with *Nla* III as the anchoring enzyme. After ligation of linkers to the *Nla* III-compatible sticky ends, the cDNA fragments were digested with the tagging enzyme BsmF I (a site present in the linkers) for 1 h at 65°C, thus generating 10 bp (base pairs) SAGE cDNA tags attached to the linkers. After blunting of the linker cDNA products with Klenow enzyme, dimerization and PCR amplification using primers directed against linker sequences, the linkers were excised by *Nla* III digestion. The released ditag was purified from the excised linkers by polyacrylamide gel electrophoresis (PAGE) and ligated to long multimers of ditags separated by *Nla* III sites. After size separation by PAGE, the 0.7- to 2-kb fraction was excised from the gel, purified, and cloned into the pZero-1.1 vector (Invitrogen). Competent TOP10 E. coli (Invitrogen) were transformed, and colonies screened by PCR for inserts using M13F and M13R primers. Positive clones were sequenced until 46,000 tags/genotype were obtained. SAGE data were analyzed using the SAGE™ software (Memorec Stoffel GmbH, Cologne, Germany), an extensive proprietary tag database that establishes automatic annotations derived from EST/genomic data and, in addition, contains 700 manually annotated tags elusive to the automated mapping. The program extracts 11 bp SAGE tags and guarantees a more reliable assignment to UniGene clusters than programs working with 10 bp tags. Proprietary filtering algorithms eliminate SAGE artifacts resulting from polymorphic tags, ribosomal RNA, linker tags and LINE/SINE tags. For comparison, all SAGE libraries were normalized to 100,000 tags. The calculation of statistical significance levels in SAGE is based on a formula developed by Audic and Claverie [50]. Significance was ascribed for p<0.01.

### Human brain samples

Human brains were collected within 80 hours of death and frozen at −80°C for biochemical analysis or paraffin-embedded for immunofluorescence (IF) staining. For histology tissue from six sporadic AD (age range 63–78, mean 69.7±6.0), six sporadic PiD (age range 62–78, mean 69.8±6.3) and eight control (CT) cases (age range 62–79, mean 70.8±6.3) was used. Frozen entorhinal cortex from six sporadic AD cases at neocortical Braak stages V–VI (age range 76–98, mean 86.3±9.0), six clinically silent cases, histologically at transentorhinal Braak stages I–II (age range 92–103, mean 99.0±5.4) and six control cases without tangles (age range 79–93, mean 85.7±6) was used for biochemical analysis. Controls were free from psychiatric, neurological or neuropathological diseases. None of the AD or PiD cases had a family history suggestive of an autosomal dominant disease.

### Antibodies

The following antibodies were used: a rabbit polyclonal antibody (Abcam #ab38148; WB (immunoblotting) 1/1000; IF (immunofluorescence) 1/200) was used to detect SFPQ. Mouse anti-glyceraldehyde-3-phosphate dehydrogenase (GAPDH; Abcam, Cambridge, MA) and a mouse anti-α-actin antibody were used as loading control (Chemicon, North Ryde, NSW, Australia). Tau13 was used to detect human tau (Abcam; #ab24634; IF 1/1000), a mouse monoclonal antibody against GFAP to stain astrocytes (Sigma, St Louis, MO; #G3893; IF 1/1000), a mouse monoclonal MAP2 antibody to stain neurons (Sigma; #M4403; IF 1/500), and mouse monoclonal antibody to detect the V5 tag (Invitrogen, IF 1/400). Secondary antibodies were coupled to Alexa-488 (Molecular Probes, USA, red, 1/200) or −555 (green, 1/200) for confocal microscopy. DAPI (4′,6-diamidino-2-phenylindole, dihydrochloride) (Molecular Probes, Invitrogen) was used for nuclear staining.

### Immunofluorescence and Western blot analysis

Immunofluorescence and Western blot analysis of murine and human tissue were done as described previously [51].

### Cell lines and immunofluorescence staining

SH-SY5Y is human neuroblastoma cell line [52]. P301L-expressing SH-SY5Y cells and mock controls were generated using lentiviral gene transfer [53]. The cells were grown in DMEM (Invitrogen) containing 10% of heat inactivated fetal bovine serum (FBS) and penicillin/streptomycin (Invitrogen) with or without blasticidin (Invitrogen) [51]. Growth arrest was achieved by incubating cells for 2 h with 10 µg/ml mitomycin C (mito C) followed by a 24 h recovery, and neuronal differentiation by incubation with 20 µM all-trans-retinoic acid for 24 h, respectively. For immunofluorescence staining, cells were plated at a density of 20.000 cells per cm^2^ on 13 mm plastic cover slips (Sarstedt, Ingle Farm, SA, Australia), followed by fixation in 4% PFA. Blocking and permeabilization was done in 10% BSA and 0.3% Triton X-100 in 0.1 M PBS for 1 h. Primary antibodies were incubated overnight at 4°C in 5% BSA, 0.1 M PBS. Secondary antibodies were coupled to Alexa-488 or -555 (Molecular Probes, USA), followed by confocal microscopy.
