# Correction: Tau-Mediated Nuclear Depletion and Cytoplasmic Accumulation of SFPQ in Alzheimer's and Pick's Disease

**DOI:** 10.1371/annotation/6650167a-7567-4c65-931f-4be7145a39fc

**Published:** 2012-09-06

**Authors:** Yazi Ke, Joe Dramiga, Ulrich Schütz, Jillian J. Kril, Lars M. Ittner, Hannsjörg Schröder, Jürgen Götz

There was an error in the first author's name. Yazi D. Ke is correct.

The correct citation is: Ke YD, Dramiga J, Schütz U, Kril JJ, Ittner LM, et al. (2012) Tau-Mediated Nuclear Depletion and Cytoplasmic Accumulation of SFPQ in Alzheimer's and Pick's Disease. PLoS ONE 7(4): e35678. doi:10.1371/journal.pone.0035678.

The Author Contribution, Performed the experiments, should list YDK JD US.

